# Patient satisfaction survey of the “Healthy Heart” pharmaceutical care service – evaluation of pharmacy labelling with pharmaceutical pictograms

**DOI:** 10.1186/s12913-023-09986-4

**Published:** 2023-09-07

**Authors:** Piotr Merks, Urszula Religioni, Miłosz Jaguszewski, Agnieszka Barańska, Agnieszka Neumann-Podczaska, Justyna Kaźmierczak, Eliza Blicharska, Katarina Fehir Šola, Regis Vaillancourt

**Affiliations:** 1 Department of Pharmacology and Clinical Pharmacology, Faculty of Medicine, Collegium Medicum, Cardinal Stefan Wyszyński University, Warsaw, Poland; 2 School of Public Health, Centre of Postgraduate Medical Education, Warsaw, Poland; 3 First Department of Cardiology, Medical University of Gdansk, Gdańsk, Poland; 4 Department of Medical Informatics and Statistics With E-Health Laboratory, Medical University of Lublin, 20-954 Lublin, Poland; 5 Department of Palliative Medicine, Poznan University of Medical Sciences, Poznan, Poland; 6 Zdrovit Sp.Z O.O, Piekary Śląskie, Poland; 7 Department of Pathobiochemistry and Interdisciplinary Applications of Ion Chromatograph, Medical University of Lublin, 1 Chodźki Str., 20-093 Lublin, Poland; 8 European Association of Employed Community Pharmacist (EPhEU), Vienna, Austria

**Keywords:** Adherence, Non-adherence, Pharmaceutical care, Pharmaceutical pictograms, Cardiovascular diseases

## Abstract

**Introduction:**

Low adherence is a major challenge in healthcare worldwide, being particularly dangerous for patients with chronic diseases, such as cardiovascular diseases and heart failure, where strict adherence is essential. Non-adherence is observed in almost half of patients, and the consequences encompass a lack of therapeutic effects, health deterioration, decreased quality of life, and even death. For cardiovascular patients, the great importance of health education and pharmaceutical education can be provided within pharmaceutical care in community pharmacies. Therefore, our study aimed at evaluating the level of satisfaction with the “Healthy Heart” pharmaceutical service, in which patients received pictograms with dosage information affixed to their medication.

**Material and methods:**

The study was designed for patients who had been prescribed an antiplatelet medication for the first time. The patients were recruited by 577 pharmacies that took part in the study after completing a special course. Ultimately, 1590 patients were enrolled in the study. The project ran from November 2019 to January 2022.

**Results:**

Most of patients had a positive attitude to the “Healthy Heart” pharmaceutical service. More than 85% of the respondents were of the opinion that the pictograms facilitated the use of the medication, and 81.7% of the respondents stated that the system of labels helped in adherence. Over 66% of the respondents thought that such labels should be included in pharmacy services, and 77.92% of the participants reported that this system of labelling medications should be offered through all pharmacies.

**Conclusions:**

Pharmaceutical labels in the pharmacists’ everyday practice can largely improve patient adherence. These efforts, provided as part of their pharmaceutical services, can have a huge influence on optimisation of patient health outcomes.

## Introduction

Non-adherence among chronic patients is one of the most serious health dangers [1, 2]. The World Health Organisation (WHO) points out that improving adherence is an essential health challenge for healthcare system worldwide [3].

Non-adherence is defined as the failure of a patient to comply with physician’s or another medical professional’s treatment recommendations [2]. It is estimated that 50% of patients do not adhere to treatment recommendations, which results in a failure to achieve expected health outcomes, deterioration in health and even possibly death [4–6].

Adherence is essential for patients with cardiovascular diseases [7, 8], the leading cause of death globally [9]. Stroke is of particular importance in this context as it is the second leading cause of death and a major cause of disability worldwide [10, 11]. European data indicates that strokes account for 8% of all deaths (2017) [12]. Studies report strong interactions between the brain and the heart. Literature reviews show both the impact of heart disease on the development of brain disease, and the effects of brain disease on the development of heart disease, with a special emphasis placed on cardiovascular complications in one-third of patients after a stroke [13–15]. It is estimated that the global lifetime risk of stroke from the age of 25 years onward is 25% [16]. A rapidly ageing society and a lifestyle contributing to cardiovascular diseases can be a factor for the prediction of an increasing share of this diagnosis in the morbidity and mortality of patients worldwide [17].

In order to prevent cardiovascular events in adult patients with a history of acute coronary syndrome or myocardial infarction and a high risk of cardiovascular events, Brilique in combination with acetylsalicylic acid (ASA) [18] can be used. Antiplatelet treatment usually lasts 12 months, but ceasing treatment can increase the number of ischaemic events, including cardiovascular death, heart attack and stroke.

Unfortunately, the research indicates a very low degree of adherence patients for therapy, staggering only about 50% [19, 20]. In this case, actions are necessary, mainly at the level of pharmaceutical care, to increase the degree of patients' use to therapeutic recommendations. One of them is the use of pharmaceutical pictograms that can significantly improve the adherence patients for therapy [21, 22].

Considering the above, the main objective of this study was to evaluate the level of satisfaction with the “Healthy Heart” pharmaceutical service, where patients received pictograms affixed to product packaging with information regarding dosage, and specially designed educational material for patients after a heart attack.

## Material and methods

### Study design

The conducted study was approved by the Bioethics Committee operating at the Ludwik Rydygier Collegium Medicum in Bydgoszcz, Nicolaus Copernicus University in Toruń of June 19, 2018 (463/2018).

Any pharmacy dispensing Brilique could participate in this study. Registration was done via the website www.uslugazdroweserce.pl. This project was conducted by Scientific Consortium Piktorex – InnoTech – UKSW sp. z.o.o.

After registration, pharmacies were directed to the training. The application for the training was done via the website of the District Pharmacy Chamber in Krakow (https://e-dukacja.pl/#/).

**Fig. 1:**
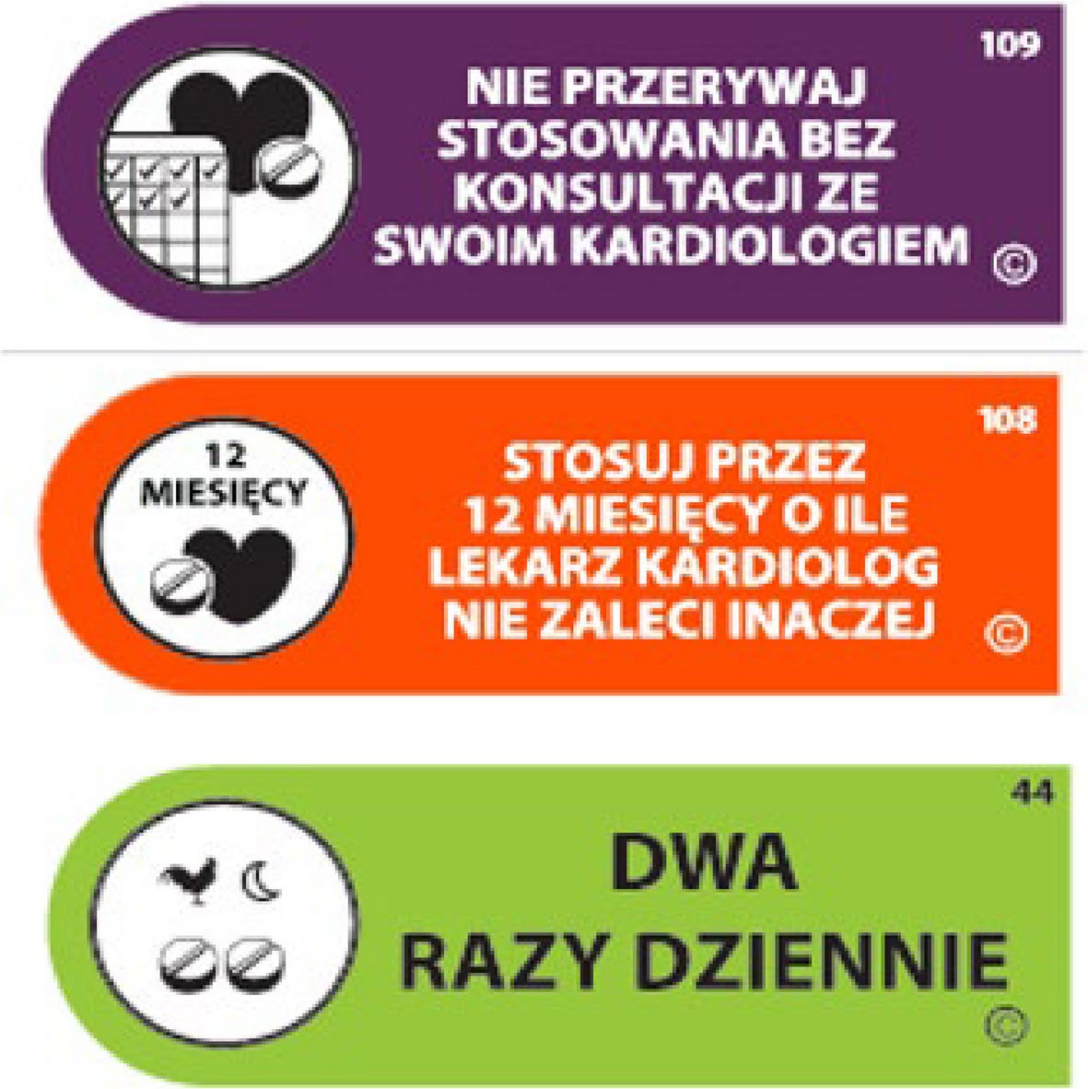
Samples of pictograms affixed to medications (in Polish) (109 – do not stop using it without consulting your cardiologist. 108 – use for 12 months, unless your cardiologist recommends otherwise. 44 – twice a day)

Having successfully completed the training and received the certificate, the pharmacies contacted the project coordinator in order to obtain educational materials for pharmacies (standard operating procedure), patient leaflets on non-adherence to post-heart attack treatment, pictograms on medications (Fig. 1), questionnaires for patients, and access to Opfarm – Soft Nova Pop Up Prompt (comorbidities screening service) that collected the data from the survey, and the software installation guide.

The project received the patronage of the Rector of the Cardinal Stefan Wyszyński University in Warsaw (UKSW), the Polish Pharmaceutical Chamber (NIA) and the Trade Union of Pharmacy Workers (ZZPF).

### Medication labelling system

Pharmaceutical labelling of medicinal products plays a key role in the process of providing essential information about their dosage and use.

Pharmaceutical labelling means providing crucial information on a given medication, mostly in the form of pictograms affixed to medicinal product packaging. This can include both descriptive and graphic information (pictograms). Labelling is affixed to the outer packaging and the medications so that patients can view them prior to using a given product.

The Individual Medication Labelling System (in Polish: System Indywidualnego Etykietowania Leków, SIEL) meets 3 primary objectives:

1. Describing and identifying a medication.
2. Contributing to achieving optimum pharmacotherapy outcomes and avoiding errors connected with the inappropriate use of medicinal products.
3. Acquiring the ability to use and store a given medication by the patient.

The most important elements and information regarding a medication should be located in a leaflet in one place and in close vicinity so that it is possible to read them at a glance. The primary elements of information for patients include:

1. General information: warnings and instructions/hints for use (if necessary).
2. Customised information for patients: dosage instructions.

The patient information leaflet should have a designated space for a machine-printed pharmaceutical label. The size of a clear font (e.g. Arial) should be at least 2 mm. The print should not fade after exposure to water or sunlight. Abbreviations and unknown expressions should not be used, particularly in the case of instructions regarding administration and dosage of medicinal products. Graphic symbols should not be used independently, but along with written instructions.

Instructions for pharmacists in training materials included the following information:

1. The Lekolepki labels should be affixed to medicinal products in pharmacies in compliance with international standards as a supplement to the basic pharmaceutical advice provided by pharmacists in pharmacies;
2. Affixing the Lekolepki labels to medicinal products is legal in the Republic of Poland;
3. The Lekolepki labels can be affixed to medication packaging anywhere, provided they do not cover:
  - the medication trade name,
  - active substance name,
  - barcode,
  - expiry date and batch number,
  - or – if possible – the manufacturer’s trade mark.

In addition, the pharmacists' training materials contained information on the Standard Operating Procedure (SOP). According to the SOP, after the patient comes to the pharmacy and shows the prescription for the drug, the pharmacist scans the barcode on the drug package. When the algorithm for testing the active substance or combination of drugs is matched, further instructions on how to proceed are displayed on the pharmacist's computer screen. The message on the screen indicates the way of proceeding for the pharmacist and offers the patient entry to the "Healthy heart" program.

### Setting

This study was conducted in community pharmacies in Poland that had registered for this project and undergone the preliminary training.

Ultimately, 577 community pharmacies were enrolled in the study and provided 1585 patient contacts. During the project, 4515 pictograms were affixed to Brilique medications.

### Measures

The research tool was a special anonymous questionnaire designed to evaluate patient satisfaction. The questionnaire was provided to people who expressed their willingness to participate in this study, after getting to know the objective and scope explained by a pharmacist. The questionnaire consisted of 9 questions regarding satisfaction with the labels affixed to the medications, along with some personal data (sex, age).

### Participants and inclusion criteria

Patients were recruited by pharmacists working in the participating community pharmacies. The Healthy Heart pharmaceutical education service supports a patient with post-acute coronary syndrome (STEMI, NSTEMI, unstable angina) who is prescribed an antiplatelet drug ticagrelor. The inclusion criterion was also the age of the patients > 18 years.

### Study length

The pilot study of the “Healthy Heart” pharmaceutical service ran between 1 November 2019 and 30 January 2022.

### Procedure – recruitment and data collection

The study comprised patients who had received their first prescription the Brilique medication, used in diseases that require an antiplatelet medication. When patients taking this group of medications visited a pharmacy, a pharmacist received a pop-up, suggesting that a given patient qualified for this study.

Ultimately, 1590 patients were enrolled in the study. The study took place according to the following Standard Operating Procedure:

1. The patient visits a pharmacy.
2. The patient provides a prescription for a medicine.
3. The pharmacist scans the barcode on the medication packaging. When the algorithm checking the active substance or the combination of medications has been confirmed, further instructions will be displayed.
4. The on-screen information will give instructions on how to offer the patient participation in the “Healthy Heart” project.

The qualification procedure for the “Healthy Heart” pharmaceutical service is very simple and looks as follows:

1. **Visiting the pharmacy**

The patient visits the pharmacy to have the prescription filled.

2. **Filling the prescription**

The pharmacist proceeds to fill the prescription.

3. **Selection of patients from the target group**

If the patient takes an anticoagulant, a pop-up is displayed and the computer system suggests the pharmacist taking a history acc. to the special protocol, and affixing 3 pictogram labels.

4. **An invitation to participate in the study**

The pharmacist asks the patient about their willingness to take part in the study, prints the consent and conducts a follow up survey on the level of satisfaction. All participants agreed to participate in the study and gave their written informed consent.

The “Healthy Heart” service consists of three main parts:

1. The patient expresses their willingness to participate in this study by signing the consent form to use their information during the study.
2. The pharmacist dispenses the antiplatelet medication packaging with 3 pictograms, one of which informs about dosage, and a leaflet with essential information on the medication. The pharmacist describes the labels and dispenses the leaflet.
3. The pharmacist contacts the patient by phone or personally in order to obtain information regarding the level of satisfaction with the tools used to provide this service.

### Data management and analysis

The collected data were entered into a spreadsheet (Excel). Data analysis was performed using Statistica v.10.

## Results

### The characteristics of the participating pharmacies

The number of participants undergoing the qualifying training for the next part of the project in the first month (December 2019) was 768. Ultimately, 941 people took part in the training (as of November 2021).

In December 2019, the number of completed tests qualifying for the project was 293, accounting for 38.15% of all the participants undergoing the training this month. At the end of the recruitment, in November 2021, this number was 3644 in total (52.49% of all the initial participants). This data means that slightly more than half of the people willing to take part in the project and undergoing the training, passed the qualifying test.

At the onset of the project, the number of pop-ups regarding Brilique was 48, with the highest number of pop-ups in July 2020 (more than 130).

In total, 577 community pharmacies (267 independent and 310 chain pharmacies) took part in the study. The largest number of pharmacies came from the following regions: Lubuskie (24.78%), Mazowieckie (14.21%) and Łódzkie (12.31%).

### The characteristics of the participating patients

1590 patients were qualified for the study, of which 67.74% were men and 32.26% were women (Table 1). The largest group of patients were aged 60 years and older (75.66%), and the smallest group were between 30–39 years of age.

**Table 1:** Characteristics of the patients (*N* = 1590)

Variable	N (%)
Sex	
Woman	513 (32.26)
Man	1077 (67.74)
Age	
30–39	27 (1.70)
40–49	99 (6.23)
50–59	261 (16.42)
> 60	1203 (75.66)

### Patient satisfaction with the service

The patients were asked if they had received written instructions regarding medications from their physician. 84.15% of the patients indicated that they had received written instructions regarding medications from their physician, and 15.85% had not received this information. Nearly 40% of the patients reported that they sometimes lost the papers with the dosages of the medications prescribed by their physician.

The patients were asked if their physician provided them with information regarding prescribed medications. Though the majority of the patients stated that their physician provided them with this information (39.06% definitely agreed, and 44.72% rather agreed with this statement), about 16% of the patients were not given this information (Table 2).

**Table 2:** Patient assessment of the labels on medications (*N* = 1590)

Question	Definitely not	Rather not	I have no opinion	Rather yes	Definitely yes
	N(%)				
Does your physician give you information about the prescribed medications?	30 (1.89)	126 (7.92)	102 (6.42)	711 (44.72)	621 (39.06)
In your opinion, do the labels facilitate the use of medications?	30 (1.89)	54 (3.40)	147 (9.25)	726 (45.66)	633 (39.81)
In your opinion, is the labelling system helpful for adherence?	6 (0.38)	57 (3.58)	228 (14.34)	657 (41.32)	642 (40.38)
In your opinion, should the labelling system be included in pharmaceutical services?	60 (3.77)	51 (3.21)	423 (26.60)	669 (42.08)	387 (24.34)
In your opinion, should pictograms be affixed upon patient request?	66 (4.15)	90 (5.66)	375 (23.58)	630 (39.62)	429 (26.98)
In your opinion, should the labelling system be offered through all pharmacies?	39 (2.45)	33 (2.08)	279 (17.55)	675 (42.45)	564 (35.47)

The vast majority of the patients were of the opinion that pictograms can facilitate the use of these products (the sum of the responses “definitely yes” and “rather yes” was more than 85%).

For 81.7% of the patients the labelling system was helpful with adherence, and 66.42% thought that it should be included in pharmaceutical services. Over 66% of the patients indicated that the pictograms should be affixed upon customer request, and 77.92% reported that this labelling system should be offered through all pharmacies.

Nearly 47% of the patients were of the opinion that all medications should have pictograms, 41.51% preferred them affixed to prescription medications, and 11.51% to OTC medications.

## Discussion

Our study showed that most of patients had a positive attitude to the “Healthy Heart” pharmaceutical service. More than 85% of the respondents were of the opinion that the pictograms facilitated the use of the medication, and 81.7% of the respondents stated that the system of labels helped in adherence. Most of patients reported that this system of labelling medications should be offered through all pharmacies.

Patients after a heart attack are a special risk group. The health consequences after a heart attack are very serious, even leading to disability and death. In addition, it should be noted that the treatment of cardiovascular diseases, such as heart attacks and strokes, is associated with high costs, e.g. due to the necessity of rehabilitation. The cost of cardiovascular diseases, including stroke, was globally about USD 863 billion, with predictions rising to USD 1.044 billion by 2030. In Poland, these costs ranged from EUR 8.2 billion to over EUR 9.6 billion for the 2015–2017 period [23]. Annual stroke-related care costs in Poland are about EUR 560 million [24]. Inpatient care costs are the largest component of direct medical stroke-related expenses [12], but other costs generated by patients should also be taken into account, such as pensions, benefits, costs of lost productivity, incapacity for work and permanent incapacity for work.

One method to improve treatments in patients after a heart attack is education. Not only do physicians play a significant role in this respect, but also pharmacists, who can use their knowledge of medications and become counsellors in pharmacotherapy. The standardisation of pharmaceutical services provided as part of pharmaceutical care are essential. Pharmaceutical care is defined as the process through which a pharmacist cooperates with a patient and other professionals in monitoring a therapeutic plan that will produce specific therapeutic outcomes for the patient. According to the Pharmaceutical Care Network Europe (PCNE), pharmaceutical care is the pharmacist's contribution to the care of individuals in order to optimise medicine use and improve health outcomes [25].

Pharmaceutical care involves three core elements:

1. Detecting real and potential drug-related problems,
2. Solving real drug-related problems,
3. Preventing drug-related problems [26].

A drug-related problem (DRP) is defined as any undesirable event experienced by a patient that involves or is suspected to involve drug therapy, and that interferes with achieving the desired goals of therapy [25]. The American Society of Health-System Pharmacists (ASHP) indicates that the most common drug-related problems are inappropriate medication, inappropriate dosage (overdosage or underdosage), non-adherence, duration of treatment too long, adverse reactions, and drug interactions [27].

Implementation of evidence-based pharmaceutical care (EBPC) is essential to avoid serious health consequences from inappropriate pharmacotherapy. A core area of pharmaceutical care is patient adherence to treatment, and the therapeutic plan is exceptionally important for patients with cardiovascular diseases [28]. For example, studies show that as adherence decreased by 10% the risk of stroke increased by 10% [29]. Adherence is all the more important in most patients with cardiovascular diseases who are elderly and with comorbidities requiring long-term treatment [28]. In addition, the amount of administered medication can impact the incidence of medication errors that may result from a missed dose, inappropriate dose [30], and drug interactions.

Despite the fact that pharmaceutical care in Poland is practised mainly in hospitals, where pharmacists pay attention to treatment optimisation and also act as health educators [31], their role in the care of outpatients is crucial [32]. Cooperation between pharmacists and attending physicians can largely improve patient health, mainly through increased adherence. Studies indicate that pharmaceutical care significantly enhances health outcomes [33, 34], as in the case of cardiovascular diseases [35, 36].

The essence of pharmaceutical care is patient-oriented care that guarantees the possibility of achieving optimum health outcomes, provided patients understand the instructions e.g. on how to properly take their medications. One solution used to increase such understanding is the use of pictograms (labels affixed to medications) that contain proper ways to take medications along with warnings concerning the medication [37, 38].

Studies show that pharmaceutical pictograms are most popular among patients with low health literacy (the ability to read healthcare information). Health literacy is the degree to which individuals have the capacity to obtain, process and understand the basic health information and services needed to make appropriate health decisions [39]. In this context, one can distinguish the ability to use medications, defined as the skill to safely and properly access, understand and act based on elementary information about medications [40]. Health literacy in European countries is estimated at 29–62%, with the lowest rate amongst those with a low financial status, low education and the elderly [41]. Low health literacy is associated with a considerably higher risk of mortality [42]. Failure to understand health-related information, such as the instructions for the use of medications, can largely increase the risk of medication errors.

In order to avoid serious health and life consequences caused by an inappropriate use of medications, it is necessary to implement pharmaceutical care services that can substantially improve and monitor health outcomes. This is particularly important in the case of limited access to physicians, observed commonly since the outbreak of the COVID-19 pandemic, and also taking into account short visit lengths that make it impossible to acquaint patients with health instructions.

Pharmacists’ knowledge allows them to help patients understand instructions regarding medications. An additional element that supports patients in remembering information about products are pictograms that can be affixed when purchasing medications. Studies indicate that this solution significantly boosts patient understanding of information. For example, a study carried out among people aged 65 years and older, mostly with elementary, low (56%) or secondary (42%) education shows that pharmaceutical pictograms considerably improve their understanding information on medications. These findings are particularly significant when taking into account the low health literacy in this group of patients [43].

Another study that included mainly people with low education, a median age of 48, civilisation diseases, and taking on average 6 medications, confirms that pictograms significantly improve understanding and adherence to pharmaceutical instructions predominantly among those aged 50 years and older and suffering from at least 3 chronic diseases [44].

Considering the above, pharmaceutical pictograms can largely prevent medication errors and thus accelerate the achievement of expected health outcomes. Pharmacist consultations on how to take medications can additionally identify an inappropriate polytherapy often resulting from multiple medical consultations, and prevent drug interactions, quitting therapy or switching from one medication to another, often without the same health effects.

## Limitations of the study

We are aware of the limitations related to the conducted study. First of all, only pharmacies that wanted to introduce a new drug service for patients took part in the study. Although the project was open to all of them, only those open to new challenges participated. Since the intervention consisted of several elements (education at the counter, pharmacy software's pop-ups, pictograms, leaflets), it would be interesting to change which of the components was most important. Interventions that contributed to the improvement in medication-taking behavior should also be measured the same as pharmacist opinions about the project. The current study design increases the external validity of the findings, thus making our approach easy to implement in standard clinical practice. Despite the fact that the quality of the pharmacist counseling was not assessed as part of the study design, the current findings still demonstrated that the current intervention can significantly improve patient compliance with medication.

## Conclusions

This study, conducted as part of the “Healthy Heart” project, shows high patient satisfaction with pharmaceutical labels. The vast majority of the patients were of the opinion that this service should be offered through all pharmacies. This information is particularly important taking into account the patients’ high non-adherence, which is associated with a considerable risk of health deterioration, namely in people after heart attack, where adherence to pharmacotherapy is a priority. Non-adherence can lead to serious health consequences, such as a deterioration in quality of life, and high costs incurred by healthcare systems.

Considering the scale of non-adherence and the relatively low level of health literacy, particularly among people with a low education and among elderly people, pharmaceutical care seems essential for patients with cardiovascular diseases, such as heart attack. These activities are aimed at reducing the risk of complications.

Due to population ageing and lifestyle, the number of cases of cardiovascular diseases has been on the rise, which will place an additional burden on healthcare systems. For this reason, the role of pharmacists in monitoring and optimising treatment should be increased, especially in Poland where pharmacists are unappreciated. Taking into account the experiences of pharmaceutical care in other European countries, the development and implementation of Good Pharmaceutical Practices in order to optimise pharmacotherapy and improve adherence will be a priority for pharmacies in Poland. Pharmaceutical labels, as elements of pharmaceutical services, and are a common solution in many countries, and are positively evaluated in numerous studies.

These activities will lead to a more appropriate use and storage of medications, and limit the risk of quitting the treatment and switching medications, which should result in better health outcomes, a higher quality of life from a better health status, and reduced costs for healthcare systems.

## Data Availability

All data are available from the corresponding author.

## Abbreviations

ASA: Acetylsalicylic acid
ASHP: The American Society of Health-System Pharmacists
DRP: Drug-related problem
EBPC: Evidence-based pharmaceutical care
PCNE: Pharmaceutical Care Network Europe
WHO: World Health Organization
